# Transcriptome Meta-Analysis Associated Targeting Hub Genes and Pathways of Drought and Salt Stress Responses in Cotton (*Gossypium hirsutum*): A Network Biology Approach

**DOI:** 10.3389/fpls.2022.818472

**Published:** 2022-04-25

**Authors:** Nasreen Bano, Shafquat Fakhrah, Chandra Sekhar Mohanty, Sumit Kumar Bag

**Affiliations:** ^1^CSIR-National Botanical Research Institute (CSIR-NBRI), Lucknow, India; ^2^Academy of Scientific and Innovative Research (AcSIR), Ghaziabad, India; ^3^Department of Botany, University of Lucknow, Lucknow, India

**Keywords:** stress, meta-analysis, cotton, hub genes, network

## Abstract

Abiotic stress tolerance is an intricate feature controlled through several genes and networks in the plant system. In abiotic stress, salt, and drought are well known to limit cotton productivity. Transcriptomics meta-analysis has arisen as a robust method to unravel the stress-responsive molecular network in crops. In order to understand drought and salt stress tolerance mechanisms, a meta-analysis of transcriptome studies is crucial. To confront these issues, here, we have given details of genes and networks associated with significant differential expression in response to salt and drought stress. The key regulatory hub genes of drought and salt stress conditions have notable associations with functional drought and salt stress-responsive (DSSR) genes. In the network study, nodulation signaling pathways 2 (*NSP2)*, Dehydration-responsive element1 D (*DRE1D*), ethylene response factor (*ERF61*), cycling DOF factor 1 (*CDF1*), and tubby like protein 3 (*TLP3*) genes in drought and tubby like protein 1 (*TLP1*), thaumatin-like proteins (*TLP*), ethylene-responsive transcription factor ERF109 (*EF109*), ETS-Related transcription Factor (*ELF4*), and *Arabidopsis thaliana* homeodomain leucine-zipper gene (*ATHB7*) genes in salt showed the significant putative functions and pathways related to providing tolerance against drought and salt stress conditions along with the significant expression values. These outcomes provide potential candidate genes for further in-depth functional studies in cotton, which could be useful for the selection of an improved genotype of *Gossypium hirsutum* against drought and salt stress conditions.

## Introduction

*Gossypium* (cotton) species is an excellent model plant system for polyploid plant study (Rong et al., 2005). The *Gossypium* genus comprises six tetraploids species and 45 diploids (Hawkins et al., 2006; Grover et al., 2015). Approximately two million years ago (MYA), allotetraploid species (*Gossypium hirsutum* and *Gossypium barbadense*) were formed from the interspecific hybridization events between *Gossypium herbaceum* (A1) or *Gossypium arboreum* (A2) and *Gossypium raimondii* (D5) or *Gossypium gossypioides* (D6) followed by polyploidization (Senchina et al., 2003). However, all the polyploid genomes are conserved in terms of gene content but they diversified at the subgenomic level (Chen et al., 2020). The most widely available commercial species of cotton is upland cotton (*G. hirsutum*) because it provides >90% production of world cotton while *G. barbadense* gives 3–4% and *G. herbaceum* and *G. arboreum* give 2% (Chen et al., 2007). *G. hirsutum* is the most crucial fiber-producing crop in the world and is grown in more than 80 countries.

Globally, India is the major producer of cotton, although yield productions are low due to crops suffering from one or more abiotic stress factors, such as salt, drought, heat, and waterlogging (Manikandan et al., 2020). Abiotic stresses are a prime factor for limiting plant growth and yield productivity due to constant climate change. Understanding the molecular aspects in plants with response to abiotic stresses is at primacy now (Ambrosino et al., 2020). These responses of plants are crucial to deal with environmental stresses in order to survive. The stress signal passes through the external environment, which continues to move toward the cell to trigger adaptive responses. Among the abiotic stress, drought and salinity show the most acute abiotic stress problems in the production of cotton in subtropic and tropic areas (Abdelraheem et al., 2021). Drought stress majorly affects plant growth and causes morphological, physiochemical, and physiological changes in plants, which hamper plant growth and productivity (Chandra et al., 2021), and salinity of soil also causes significant loss in the production of cotton (Manikandan et al., 2020). Both drought and salt stress have negative impacts in all the stages of growth and can reduce cotton yield by up to 50–67% (Dabbert and Gore, 2014; Ullah et al., 2017; Abdelraheem et al., 2019, 2021). Thus, an understanding of the genomic level of cotton’s response to various environmental stresses would give details on how to improve its genetic makeup with increased resistance to salt and drought stress. The availability of a large amount of transcriptome dataset deposition in Sequence Read Archive (SRA) provides an opportunity to accomplish a comprehensive gene network study (Liao et al., 2003). Understanding these gene networks in the biological insights regarding abiotic stress response in plants is quite challenging (Hartwell et al., 1999; Cramer et al., 2011; Todaka et al., 2012).

For gene regulation and construction of gene networks, transcriptome data is important for functional annotation and discovery of putative genes (Reményi et al., 2004; Ma et al., 2009). Using the genes that were expressed in transcriptome data, gene networks are built based on similarity (correlation) in gene expression (coexpression). The co-expression networks (CNs) of candidate genes help in the study of expression patterns across multiple stress conditions among a large number of genes (Merico et al., 2009; Serin et al., 2016). The Pearson correlation coefficient (PCC) calculated for the similarity score of CNs from genes with significant expression levels, such as sets of genes that have immensely correlated scores tend to group a small module. These correlated genes could be involved in associated signaling pathways and similar functions. Therefore, unknown or poorly characterized co-expressed genes in a module can be predicted by using genes of known function in certain biological processes (Mochida et al., 2011; Rhee and Mutwil, 2014). In CNs of genes, transcription factor hubs are possible contenders for gene regulatory function. Transcription factors help to suggest the co-regulation of subsets of genes that are present in the same co-functionality or signal transduction pathways during the biological processes. At the transcriptional level, co-expression of genes relies upon the promoter region with similar *cis*-regulatory elements. So, co-expression profiles of genes are integrated into *cis*-motifs in the respective promoter sequences of a module, which give insights into the regulation of coordinated genes in CNs (Ma and Bohnert, 2007; Todaka et al., 2012; Sharma et al., 2013; Wilkins et al., 2016).

High-throughput comprehensive analysis of expressed genes has become the important approach for showing the putative genes, finding *cis*-regulatory motifs, predicting gene function, and describing gene regulatory networks (Goda et al., 2008; Brady and Provart, 2009; Mochida et al., 2011). Due to the availability of large-scale transcriptome data in the public domain, we have been able to construct biological networks and pathways. Much effort has been done to make use of co-expression analysis of transcriptome data. These studies used several approaches to find genes and predict the function of genes (Aoki et al., 2007). Later, these pathways and networks have been used to understand functional interaction amongst genes (Moreno-Risueno et al., 2010).

The large-scale availability of transcriptome data in NCBI has given us the chance to build a co-expression network for *Gossypium* transcriptome in different abiotic stress conditions. These networks might be used to determine the genes that have a significant role in salt and drought stress conditions in specific gene regulatory networks. In this study, we analyzed all the available transcriptome data in NCBI related to salt and drought stress till January 2021 in different tissues and cultivars. For the construction of the co-expression network of cotton, PCC was calculated for drought and salt stress conditions in different tissues (root, leaf, and seed) along with biological processes. Moreover, hub genes were identified from co-expressed modules and also retrieved their functional enrichment and pathways and these identified hub genes were also characterized through expression profiles. These investigations showed that a co-expression module study enables to find the putative genes that could be suitable targets for crop improvement approaches aimed to protect the cotton from drought and salt stress conditions.

## Materials and Methods

### Data Collection

A total of 118 (SRA accession number = SRP242577, SRP043419, SRP228010, SRP157859, and SRP192537) and 33 (SRA accession number = SRP192537 and SRP166405) RNA-seq datasets of treated and untreated cotton samples were retrieved from the NCBI SRA database till January 2021 using specific keywords “*G. hirsutum*” and individual abiotic stress. i.e., “salinity and drought.” These datasets were extracted from the leaf, root, and seed tissues, and 4 cultivars (TM1, TM2, H15, and ZM12) in salinity and drought stress datasets were extracted from the leaf and root tissue that includes TM1 culture.

### Processing for Salt and Drought Stress Responsive Genes Identification

The parameters for the dataset taken into consideration for further study were the experiments which were conducted for drought and salinity treatment of cotton with at least two replicates (Walia et al., 2005, 2007, 2009; Jain et al., 2007; Hu et al., 2009; Mustroph et al., 2010). In total, our final meta-datasets comprised 151 RNA-seq representing 33 droughts and 118 salt-treated in 7 experiments as summarized in Supplementary Table 1. The quality control of these raw fastq datasets was processed by the Trimmomatic tool (Bolger et al., 2014) with average quality of 20 per base and adapter trimming was also done where it was required. Filtered reads were mapped on the *G. hirsutum* genome (Wang M. et al., 2019) by using a STAR aligner (Dobin et al., 2013) with the default parameter. The quantification of differentially expressed genes (DEGs) was done through cuffdiff in Cufflinks version 2.2.1 software (Trapnell et al., 2012) with *p*-values ≤ 0.05, and log2fold-change (log2FC) values ≥ 2 for upregulated and ≤ −2 for downregulated genes. The top-ranked significant DEGs were compared in different tissue under drought and salt stress conditions. Unbiased clustering of drought and salt stress-responsive (DSSR) genes from different tissues with their log2FC were displayed through heatmap using the “Pheatmap” package function in R with normalized scale and euclidean distance method.

### Gene Co-expression Network Construction for Salt and Drought Stress Dataset

Drought and salt stress-responsive genes were selected based on their differential expression in several experiments. Each tissue with higher differential expression was considered a reference in DSSR-GCN (gene co-expression network). The DSSR-GCN was constructed by the FPKM along with < 0.05 *p*-values. The ‘‘expression correlation networks’’ module in Cytoscape version 3.8^1^ (Smoot et al., 2011) was used to construct the network. This module calculated a positive PCC (*r* ≥ 0.95) among the interacting members of the network. The co-expressed genes were visualized through Cytoscape.

### Functional Enrichment Analysis of Co-expressed Genes

To identify functionally grouped GO and pathway, the ClueGo module in Cytoscape was used (Bindea et al., 2009). Using ClueGo, a biological process enrichment analysis of co-expressed genes in up and downregulated genes was performed in root and leaf tissue of drought and root, leaf, and seed tissues of salt stress datasets. Moreover, common enriched pathways between drought and salt were identified and represented through the EnrichmentMap module in Cytoscape. Node color shows the normalized enrichment score (NES) from the core enrichment genes. A positive score (red) shows gene set enrichment at the top of the ranked list a negative score (blue) indicates gene set enrichment at the bottom of the ranked list. The color of the border represents the *p*-value of the pathway enrichment from gene set enrichment analysis (GSEA). The node size shows the number (setSize) of DEGs identified in the pathways.

### Identification of Hub Genes

In a co-expression network, each edge shows an interaction between genes and the node shows a gene. We have estimated closeness connectivity, betweenness centrality (Cb), eccentricity, and degree centrality (k) using the network analyzer (Doncheva et al., 2012) plugin in Cytoscape software. Identification of hub genes was done based on connection or degree or they were shared in DSSR-GCN. Highly connected genes that have a high correlation with other genes in the network are known as hub genes. Any alteration in hub genes expression could lead to affect the major part of the network. Rapid information can be transferred in the network by the genes that have high connectivity compared to the other genes. Significantly highly expressed top-ranked genes that have a higher betweenness centrality value and degree of connectivity (k) were taken as hub genes.

Each node is allotted by a specific value based on links held through it, known as degree centrality (k). The degree helps in estimating the significance of node to control the network and it demonstrates the number of interactions retrained through a node in the network with other nodes.


(1) k=Σ⁢wvεKu⁢(u,v)

Where K_*u*_ denotes the node-set comprising entire neighbors of node u, and w (u,v) indicates the edge weight which connects node v with node u.

The betweenness centrality describes how often a node falls on the shortest path with other nodes in its neighborhood.

Betweenness centrality (Cb) measures how many times a node falls on the shortest path with other neighboring nodes. It refers to the ability of a node to control information flow and signal processing in the network.


(2) $$\text{cb}=\sum_{\text{k}\neq \text{u}\neq \text{f}}\frac{\text{fp}\left(\text{k,u,f}\right)}{\text{p}\left(\text{k,f}\right)}$$

Where, p (k,u,f) represents the number of interactions from node k to node f that pass through node u, whereas p (k,f) shows the total number of shortest interactions between nodes k and f (Prasad et al., 2020).

### Gene Ontology, Pathway Enrichment, and *Cis*-Regulatory Element Analysis of Identified Hub Genes

For extensively analyzing the functional annotations of DEGs, we used the GO analysis toolkit for the agricultural community (agriGO version 2) (Tian et al., 2017). The gene ontology provided the annotations at the cellular level, molecular level, and biological level. The GO graph was generated in RStudio using the ggplot package. Further, the functions of DSSR hub genes were confirmed through the GeneMANIA server (Warde-Farley et al., 2010), which predicts the functions of gene sets. The functional study was performed at a 0.05 *p-*value cutoff, with the *Arabidopsis thaliana* database as a reference. For KEGG pathways analysis was employed using clusterProfiler package in RStudio with the number of permutation (nPerm) set to 10,000, org.At.tair.db and pAjustMethod set to “none.” *P*-value ≤ 0.05 was considered for KEGG pathways. The PlantCARE database (Lescot et al., 2002) was searched for *cis*-regulatory elements in the 2 kb sequences upstream of identified hub genes identified by using the “Signal Scan Search” program.

### RNA Isolation, cDNA Preparation, and Quantitative Real-Time PCR Validation

To check the amplification of the key hub genes of cotton (*G. hirsutum*), plants were grown in the growth chamber under a controlled temperature of 30–37^°^C with normal photoperiodic conditions. The seedlings were grown in separate triplicate pots for 1 month, stress treatment was given with, 20% PEG 8,000 (approx 300 ml/kg of soil) in the pots for the drought stress (Patade et al., 2009) and NaCl (300-mM) for salt stress (Li et al., 2017). One time stress treatment was provided to each pot and then after intervals of 12, 24, 48, and 72 h RNA was isolated following the protocol (spectrum™ Plant Total RNA Isolation Kit). Once the RNA was isolated, cDNA was prepared (1 μg/μl) with verso cDNA synthesis kit (Thermo scientific). The amplification of the four putative genes for drought and four genes for salt stress was designed with primer3 Plus and checked with Quantitative Real-Time PCR (qRT-PCR) (HiMedia Insta Q 48 M4). The reaction was prepared with 10 μl of fast the SYBER™ green master mix (Applied Biosystem) and 1 μl of 100 ng cDNA adding 5 picomoles (forward and reverse) primers in a total 20 μl reaction. Ubiquitin was taken as internal control and the program for qRT-PCR was set at 95?C for 2 min, followed by cycling for 40 cycles of denaturation at 95^°^C for 15 s, annealing at 60 C for 1 min, and extension at 72 C for 30 s. Relative expression of the employed genes was calculated with mean ± *SD* of biological triplicate samples by the 2^^–ΔΔct^ method (Livak and Schmittgen, 2001).

### Statistical Analysis

The statistical analysis of qRT-PCR was carried out, using GraphPad Prism version 8.4.3 software, with two-tailed Student’s *t*-test in triplicate sample repeats.

## Results

### Comprehensive Differentially Expressed Genes of Drought and Salt Stress-Responsive Genes in Cotton

A total of 2 SRA accessions (SRP166405 and SRP192537) of drought and 5 SRA accessions (SRP242577, SRP043419, SRP228010, SRP157859, and SRP192537) of salt containing 33 and 118 fastq files were processed through the Trimmomatic tool. These RNA-Seq data included root, leaf, and seed of different cultivars. As a result, 2,168,052,606 clean reads with an average length of 277 bp were produced in salt stress data and 776,795,545 clean reads with an average length of 293 bp were generated in drought stress after removing the adapter sequences and low-quality reads (Table 1). The clean reads were then mapped to the *G. hirsutum* (cotton) genome, 1,960,725,723 (90.43%) reads in salt (Supplementary Table 1A) and 722984665 (93.06%) reads in drought (Supplementary Table 1B) were mapped to the reference cotton genome.

**TABLE 1A:** Top 86 hub genes based on the topological properties in co-expression network of DEGs in drought stress condition, and arranged on the basis of the decreasing degree of connectivity value.

S. No.	Gene ids	Hub genes	Hub genes descriptions	Degree	Betweenness centrality
1	Ghir_A12G023480	SMO11	Methylsterol monooxygenase 1-1	81	0.025425
2	Ghir_A06G017350	PYL6	Abscisic acid receptor PYL6	80	0.038656
3	Ghir_D09G014940	MYB36	Transcription factor MYB36	80	0.019473
4	Ghir_A09G015810	ROC2	31 kDa ribonucleoprotein	80	0.019473
5	Ghir_A06G022530	Y5780	BTB/POZ domain-containing protein	80	0.019473
6	Ghir_A13G022680	PTR51	Protein NRT1/PTR FAMILY 7.1	80	0.019473
7	Ghir_A03G015280	HEBP2	Heme-binding protein 2	78	0.015231
8	Ghir_D12G013730	USPAL	Universal stress protein A-like protein	78	0.015231
9	Ghir_A03G011520	NAC29	NAC transcription factor 29	76	0.011579
10	Ghir_D04G005670	DOX1	Alpha-dioxygenase 1	76	0.011579
11	Ghir_A06G000190	EXL3	GDSL esterase/lipase EXL3	70	0.029445
12	Ghir_D08G017530	BAMO	Beta-amyrin 11-oxidase	68	0.02602
13	Ghir_A11G002550	ERF61	Ethylene-responsive transcription factor ERF061	58	0.02197
14	Ghir_D06G015630	NCED5	Probable 9-cis-epoxycarotenoid dioxygenase NCED5	58	0.02197
15	Ghir_D11G000750	OLE16	Oleosin 16 kDa	57	0.01971
16	Ghir_D10G012460	BBE8	Berberine bridge enzyme-like 8	57	0.01971
17	Ghir_D10G009660	C84A1	Cytochrome P450 84A1	56	0.024787
18	Ghir_A04G015950	CDF1	Cyclic dof factor 1	56	0.024787
19	Ghir_D05G028710	LNK2	Protein LNK2	56	0.024787
20	Ghir_A09G019200	P2C08	Probable protein phosphatase 2C 8	56	0.022376
21	Ghir_A02G015430	MET1	Protein methyltransferase 1	56	0.015322
22	Ghir_A12G000840	GRXS9	Monothiol glutaredoxin-S9	56	0.015322
23	Ghir_A01G009970	LOG1	Cytokinin riboside 5’-monophosphate phosphoribohydrolase LOG1	56	0.014792
24	Ghir_A03G006810	PMEI7	Pectinesterase inhibitor 7	55	0.031033
25	Ghir_A11G013200	LEA3	Late embryogenesis abundant protein 3	55	0.01701
26	Ghir_A08G021170	NSP2	Nodulation-signaling pathway 2 protein	55	0.010456
27	Ghir_A08G022620	LTL1	GDSL esterase/lipase LTL1	55	0.010456
28	Ghir_D08G007420	ALMTC	Aluminum-activated malate transporter 12	54	0.029337
29	Ghir_A09G016950	RVE8	Protein REVEILLE 8	53	0.028304
30	Ghir_D05G026820	BCAT2	Branched-chain-amino-acid aminotransferase 2	52	0.025799
31	Ghir_A12G001090	TSPO	Translocator protein homolog	52	0.024852
32	Ghir_A12G027100	LTI65	Low-temperature-induced 65 kDa protein	52	0.024852
33	Ghir_A06G013500	SBT56	Subtilisin-like protease SBT5.6	52	0.014369
34	Ghir_A08G014510	EXPB2	Putative expansin-B2	52	0.014369
35	Ghir_D05G024500	CHSY	Chalcone synthase	52	0.014369
36	Ghir_D05G028190	TLP3	Tubby-like F-box protein 3	52	0.014369
37	Ghir_D01G009330	NPH3	Coleoptile phototropism protein 1	51	0.021859
38	Ghir_D08G010410	TAT2	Probable aminotransferase TAT2	50	0.025382
39	Ghir_D03G009030	GUN6	Endoglucanase 6	50	0.025352
40	Ghir_D09G016760	AMYG	Glucoamylase ARB_02327-1	50	0.019761
41	Ghir_A06G001560	PLP2	Patatin-like protein 2	50	0.019174
42	Ghir_A12G014930	EXPB3	Expansin-B3	50	0.01628
43	Ghir_D05G000070	RPB7	DNA-directed RNA polymerase II subunit RPB7	50	0.010994
44	Ghir_A03G022170	DNJ11	Chaperone protein dnaJ 11	50	0.007053
45	Ghir_D12G014830	MYB15	Transcription factor MYB15	50	0.007053
46	Ghir_D02G016110	PLY5	Probable pectate lyase 5	50	0.007053
47	Ghir_D08G018040	EXLB1	Expansin-like B1	48	0.019743
48	Ghir_D02G016770	PGLR4	Polygalacturonase	42	0.053756
49	Ghir_A05G024360	CML44	Probable calcium-binding protein CML44	41	0.029242
50	Ghir_D12G012000	AHL9	AT-hook motif nuclear-localized protein 9	40	0.049587
51	Ghir_A07G024540	SUOX	Sulfite oxidase	40	0.025008
52	Ghir_A01G005030	CLE5	CLAVATA3/ESR (CLE)-related protein 5	40	0.010874
53	Ghir_D11G026490	GL114	Germin-like protein subfamily 1 member 14	40	0.010874
54	Ghir_A12G002390	EF109	Ethylene-responsive transcription factor ERF109	40	0.010874
55	Ghir_A05G014280	DNAJ	Chaperone protein DnaJ	40	0.010874
56	Ghir_D12G020120	BT1	BTB/POZ and TAZ domain-containing protein 1	40	0.010874
57	Ghir_D05G015970	CLE6	CLAVATA3/ESR (CLE)-related protein 6	40	0.010874
58	Ghir_A09G007960	HFB2A	Heat stress transcription factor B-2a	40	0.010874
59	Ghir_A09G014950	INV1	Beta-fructofuranosidase, insoluble isoenzyme 1	36	0.027235
60	Ghir_A04G002520	CARP1	Calcium-binding protein KRP1	36	0.027235
61	Ghir_A13G016010	NCED1	9-cis-epoxycarotenoid dioxygenase NCED1	35	0.024129
62	Ghir_A08G021330	ASPRX	Aspartic proteinase	35	0.022404
63	Ghir_A09G011440	ORG2	Transcription factor ORG2	35	0.015993
64	Ghir_D11G013780	WTR31	WAT1-related protein	35	0.015102
65	Ghir_A05G035540	PRR73	Two-component response regulator-like PRR73	35	0.012788
66	Ghir_A12G019880	BT2	BTB/POZ and TAZ domain-containing protein 2	35	0.002076
67	Ghir_D03G004830	ABR1	Ethylene-responsive transcription factor ABR1	34	0.021663
68	Ghir_A10G001190	CRK10	Cysteine-rich receptor-like protein kinase 10	34	0.021663
69	Ghir_A09G012520	LCMT1	Leucine carboxyl methyltransferase 1 homolog	32	0.029281
70	Ghir_D11G001290	WRK27	Probable WRKY transcription factor 27	32	0.018759
71	Ghir_A03G002790	HSP83	Heat shock protein 83	32	0.018297
72	Ghir_D11G026510	GL117	Germin-like protein subfamily 1 member 17	32	0.013422
73	Ghir_A09G022540	PER1	Cationic peroxidase 1	31	0.025053
74	Ghir_A09G004700	DRE1D	Dehydration-responsive element-binding protein 1D	31	0.025053
75	Ghir_A09G017430	DIOX5	Probable 2-oxoglutarate-dependent dioxygenase	31	0.022918
76	Ghir_D10G003580	CLE1	CLAVATA3/ESR (CLE)-related protein 1	31	0.022535
77	Ghir_A02G001990	CASL1	Cannabidiolic acid synthase-like 1	31	0.022535
78	Ghir_A10G011670	HFA6B	Heat stress transcription factor A-6b	31	0.014
79	Ghir_A10G021880	ASP	21 kDa seed protein	31	0.012239
80	Ghir_A02G019210	TLP5	Tubby-like F-box protein 5	31	0.010166
81	Ghir_A01G003470	F6H1	Feruloyl CoA ortho-hydroxylase 1	30	0.071444
82	Ghir_D10G006510	CRR38	Cysteine-rich repeat secretory protein 38	30	0.067497
83	Ghir_D12G008960	E131	Glucan endo-1,3-beta-glucosidase 1	30	0.062128
84	Ghir_D10G005340	Y1561	Probable LRR receptor-like serine/threonine-protein kinase	30	0.031079
85	Ghir_D10G012300	NLTP2	Non-specific lipid-transfer protein	30	0.031079
86	Ghir_A07G003900	TAR4	Tryptophan aminotransferase-related protein 4	30	0.00733

**TABLE 1B:** Top 61 hub genes based on the topological properties in co-expression network of DEGs in salt stress condition, and arranged on the basis of the decreasing degree of connectivity value.

S. No.	Gene IDs	Hub genes	Hub genes descriptions	Degree	Betweenness centrality
1	Ghir_A09G015330	TLP1	Thaumatin-like protein 1	97	0.007886
2	Ghir_D03G016690	TLP	Thaumatin-like protein	97	0.007886
3	Ghir_D07G020080	WRKY6	WRKY transcription factor 6	94	0.007886
4	Ghir_A05G033720	2MMP	Metalloendoproteinase 2-MMP	94	0.007886
5	Ghir_A10G024010	ELF4	Protein EARLY FLOWERING 4	87	0.018944
6	Ghir_D02G009420	PLP2	Patatin-like protein 2	86	0.021816
7	Ghir_D11G022440	IST1L	IST1-like protein	85	0.01222
8	Ghir_D11G019230	AATP1	AAA-ATPase	85	0.01222
9	Ghir_D11G023090	PER53	Peroxidase 53	85	0.01222
10	Ghir_A10G008980	M3K17	Mitogen-activated protein kinase kinasekinase 17	84	0.020294
11	Ghir_A13G003810	EF109	Ethylene-responsive transcription factor ERF109	83	0.006739
12	Ghir_D09G018270	BHLH126	Transcription factor bHLH126	83	0.006739
13	Ghir_D07G005900	MASY	Malate synthase	82	0.004228
14	Ghir_D03G005410	EIX2	Receptor-like protein EIX2	82	0.004228
15	Ghir_D04G016980	GGP5	Gamma-glutamyl peptidase 5	82	0.004228
16	Ghir_A12G017920	CXE6	Probable carboxylesterase 6	82	0.004228
17	Ghir_D08G002910	DXS2	Probable 1-deoxy-D-xylulose-5-phosphate synthase 2	82	0.004228
18	Ghir_D12G013400	NIMI1	Protein NIM1-INTERACTING 1	82	0.004228
19	Ghir_D04G013670	LAC7	Laccase-7	80	0.002337
20	Ghir_D11G000750	OLE16	Oleosin 16 kDa	80	0.041127
21	Ghir_D04G007930	NAC83	NAC domain-containing protein 83	79	0.017827
22	Ghir_D10G012300	NLTP2	Non-specific lipid-transfer protein	79	0.001434
23	Ghir_A04G016310	DCS2	(+)-delta-cadinene synthase isozyme XC14	79	0.001434
24	Ghir_D05G020420	YODA	Mitogen-activated protein kinase kinasekinase YODA	79	0.001434
25	Ghir_A12G008500	XTHB	Probable xyloglucan endotransglucosylase/hydrolase protein B	79	0.03825
26	Ghir_A08G012870	GPAT3	Probable glycerol-3-phosphate acyltransferase 3	78	6.38E-04
27	Ghir_D11G010670	ATHB7	Homeobox-leucine zipper protein ATHB-7	78	6.38E-04
28	Ghir_A09G012630	FAB1D	Putative 1-phosphatidylinositol-3-phosphate 5-kinase FAB1D	75	0.016174
29	Ghir_D06G015630	NCED5	Probable 9-cis-epoxycarotenoid dioxygenase NCED5	75	0.016174
30	Ghir_A13G021930	CAS2	Cycloartenol synthase 2	74	0.013795
31	Ghir_A11G002550	ERF61	Ethylene-responsive transcription factor ERF061	74	0.013795
32	Ghir_D08G004260	AOC	Allene oxide cyclase	73	1.27E-05
33	Ghir_A11G022360	bHLH25	Transcription factor bHLH25	72	0.014805
34	Ghir_A12G017500	CDF1	Cyclic dof factor 1	69	0.020647
35	Ghir_A09G025580	GOLS2	Galactinol synthase 2	67	0.017988
36	Ghir_D04G011670	ATL32	RING-H2 finger protein ATL32	61	0.024505
37	Ghir_A03G003840	NRT21	High-affinity nitrate transporter 2.1	61	0.014908
38	Ghir_D11G018910	BBX19	B-box zinc finger protein 19	60	0.016913
39	Ghir_D02G019360	MIP1B	B-box domain protein 31	59	0.013115
40	Ghir_D11G025000	PER28	Peroxidase 28	59	0.013115
41	Ghir_A05G016820	GES	(E,E)-geranyllinalool synthase	55	0.00299
42	Ghir_A09G022540	PER1	Cationic peroxidase 1	55	1.22E-04
43	Ghir_D01G009330	NPH3	Coleoptile phototropism protein 1	54	0.013919
44	Ghir_A05G017210	MFT	Protein MOTHER of FT and TFL1	52	0.01097
45	Ghir_D09G004350	DRE1D	Dehydration-responsive element-binding protein 1D	50	0.045383
46	Ghir_A01G019420	DCS3	(+)-delta-cadinene synthase isozyme A	44	0.002148
47	Ghir_D09G018710	P2C08	Probable protein phosphatase 2C 8	44	0.026726
48	Ghir_A11G029200	PME41	Probable pectinesterase/pectinesterase inhibitor 41	44	0.013211
49	Ghir_D05G007260	ORG2	Transcription factor ORG2	44	0.011147
50	Ghir_A11G002140	EXPA7	Expansin-A7	43	0.018797
51	Ghir_A11G015580	CB13	Chlorophyll a-b binding protein 8	43	0.010115
52	Ghir_A03G006840	21KD	21 kDa protein	43	0.010115
53	Ghir_D05G019060	SNAK2	Snakin-2	42	0.017728
54	Ghir_A02G007670	CAO	Chlorophyllide a oxygenase	42	0.01713
55	Ghir_D13G006460	COL12	Zinc finger protein CONSTANS-LIKE 12	40	0.012856
56	Ghir_A03G018530	AB19B	ABC transporter B family member 19	39	0.010653
57	Ghir_A08G007360	ALMT8	Aluminum-activated malate transporter 8	39	0.010653
58	Ghir_D13G016910	GSTXA	Probable glutathione S-transferaseparA	35	0.01475
59	Ghir_D06G014820	SAG12	Senescence-specific cysteine protease SAG12	32	0.01092
60	Ghir_D11G016500	PRF1	36.4 kDa proline-rich protein	31	0.009919
61	Ghir_D12G000990	SRC2	Protein SRC2	31	0.009919

**FIGURE 1:**
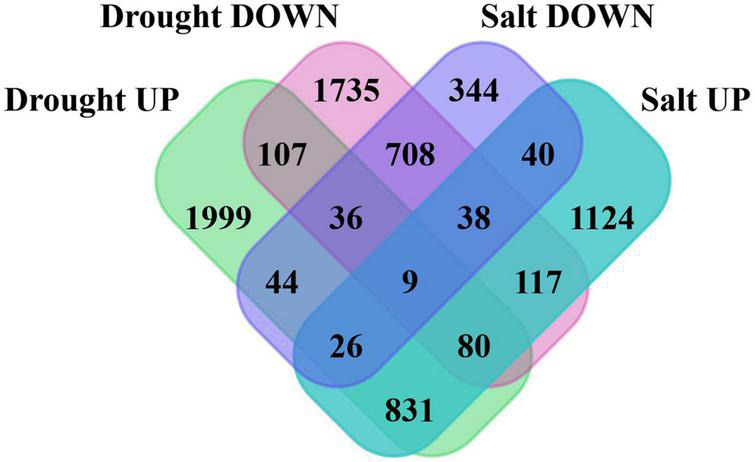
Venn diagram showing the number of shared and unique genes in differentially expressed genes (DEGs) of drought and salt stress data. In order to make drought and salt stress-responsive (DSSR) genes set compendium, we focused on 3,841 and 1,508 DSSR uniquely differentially expressed genes in drought and salt.

Further, 5,962 and 3,510 differentially expressed genes (DEGs) were identified in drought and salt stress data. Total 3,132 and 2,830 up and downregulated genes in drought and 2,265 and 1,245 up and downregulated genes (*P*-value < 0.05 and log2fold change > 2) in salt stress data. To make the summary of DSSR genes, which were expressed exclusively in drought or salt, DEGs were compared and found that 3,841 were unique in drought and 1,508 were unique in salt stress data (Figure 1). The unbiased clustering of all significant DEGs of drought (leaf and root) and salt (leaf, root, and seed) stress-responsive genes from different tissues with their expression values (log2FC) have shown through heatmap (Supplementary Figure 1) and co-existed differentially expressed genes among drought root, salt root, drought leaf, salt leaf, and salt seed have been presented in Supplementary Figure 2.

### Generation of Cotton-Drought and Salt Stress-Responsive Gene Network by Using Transcriptome Study

**FIGURE 2:**
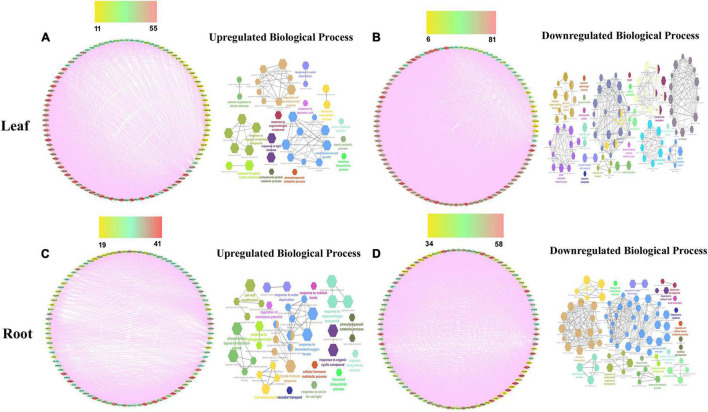
The drought-responsive gene network analysis of the top 100 upregulated **(A,C)** genes with their biological processes **(B,D)** in leaf and root tissues. Scales are showing from lower to higher degree connectivity.

**FIGURE 3:**
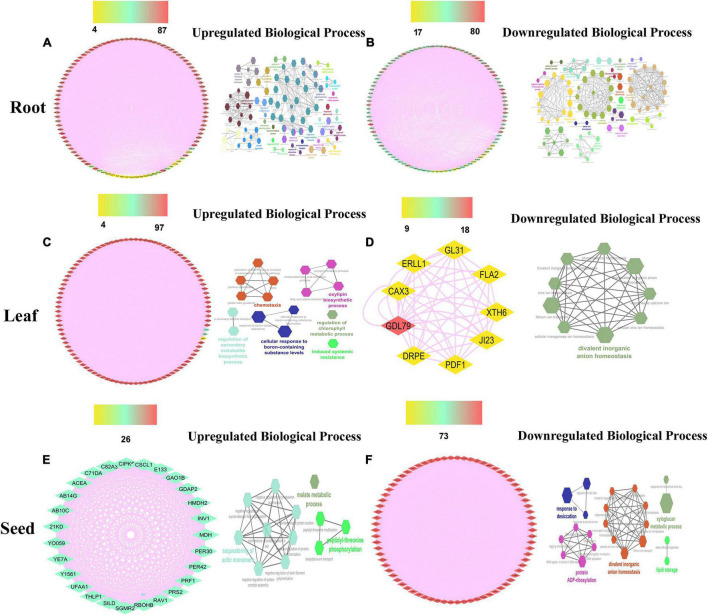
The salt responsive genes network analysis of the top 100 upregulated **(A,C,E)** genes with their biological processes **(B,D,F)** in leaf, root, and seed tissues. Scales are showing from lower to higher degree connectivity.

To build the cotton-DSSR gene network, we have used the analyzed transcriptome data of cotton in two abiotic stress (drought and salt) conditions in different tissues. The protein-protein interaction network was constructed in a circular layout after co-expression calculation using the Expression Correlation module in the Cytoscape tool. Further, among the analyzed transcriptome data, the top 100 DEGs genes with FPKM values and significant *p-*value (< 0.05) were selected from each tissue for further co-expression network analysis using the PCC method. Based on correlation score calculation drought-responsive gene co-expression network (GCN) was generated which comprised 100 nodes connected by 1,704 and 2,747 co-expressed edges with minimum network density (ND) 0.34 and 0.55 in up- and downregulated genes of leaf tissue at PCC ≥ 0.95. Similarly, drought-responsive GCN of root tissue was generated which comprised 100 nodes connected by 1,505 and 2,314 co-expressed edges with minimum ND 0.30 and 0.46 in up and downregulated genes. Moreover, the salt responsive GCN of leaf tissue was generated which comprised 98 and 9 nodes connected by 4,565 and 45 co-expressed edges in up and downregulated genes, whereas 100 nodes connected with 3,252 and 2,604 co-expressed edges with minimum ND 0.65 and 0.52 in up- and down-regulated genes of salt root tissue. Similarly, salt responsive GCN of seed tissue was generated which comprised 27 and 74 nodes connected with 351 and 2,701 co-expressed edges in up and downregulated genes. These co-expressed genes were further used for network analysis of significant DEGs for drought and salt stress conditions. For this we have estimated closeness connectivity, betweenness centrality (Cb), eccentricity, and degree centrality (k) using network analyzer plugin in Cytoscape software and circular layout obtained network for drought (Figures 2A,C) and salt stress dataset at different tissue levels (Figures 3A,C,E). The genes are ranked based on betweenness centrality scores and degree connectivity in drought (Supplementary Table 2A) and salt stress dataset (Supplementary Table 2B). Among these, *SBT, EXPB2, CHS, TLP3*, and *CML44* have a high degree and betweenness centrality value in the drought stress dataset while *WRKY6, ELF4, TLP, EXLB1, AATP1*, and *PER53* comprises high degree and betweenness centrality values in the salt stress.

To acquire an in-depth understanding of the drought and salt stress-responsive gene network, biological processes analysis has been applied on top selected differentially expressed genes using the ClueGo module with a *p*-value of 0.05 plugin in Cytoscape. The biological functions in drought stress conditions were shown in Figures 2B,D and salt stress conditions were shown in Figures 3B,D,F in different tissue levels. Their detailed functional enrichments are shown in Supplementary Table 3A for drought and Supplementary Table 3B for salt datasets. In drought stress, the interacting genes in biological process (BP) were mainly enriched in the response to water deprivation, cellular response to abiotic stimulus, flavonoid biosynthetic process, phenylpropanoid metabolic process, response to jasmonic acid, and trehalose biosynthetic process in upregulated (Figure 2B) genes of leaf tissue while, flavonoid biosynthetic process, response to decreased oxygen levels, response to hydrogen peroxide and response to water deprivation in upregulated (Figure 2D) genes of root tissue. Moreover, in salt stress condition, upregulated BP were enriched with cellular response to abiotic stimulus, flavonoid metabolic process, isoprenoid metabolic process, hyperosmotic salinity response, response to abscisic acid, response to decreased oxygen levels, response to extracellular stimulus, and response to jasmonic acid in root tissue (Figure 3B) while regulation of secondary metabolite biosynthetic process, induced systemic resistance, oxylipin biosynthetic process and cellular response to boron-containing substance levels were upregulated BP in leaf tissue (Figure 2D). Moreover, upregulated BP in seed tissue were malate metabolic process peptidyl-threonine phosphorylation, basipetalauxin transport, peptidyl-threonine phosphorylation, and maintenance of protein location in the cell (Figure 3F). The co-expression network and their functional enrichment study highlighted the key role of these putative genes in drought and salt stress responses in both (leaf and root) tissues except seed. Therefore seed tissue was dropped from further in-depth studies.

### Functional and Biological Pathways Investigation of Drought and Salt Stress-Responsive Genes

The gene Ontology is used to assign putative functions to all identified DEGs into three categories: biological processes (BP), cellular components (CC), and molecular functions (MF). Significantly differentially expressed genes were taken for further functional and biological pathways investigation. In drought stress conditions, gene ontology analysis in both the tissues (leaf and root) showed a significant presence of peroxidase activity, carboxylesterase, and petinesterase activity in molecular functions (MF). Biological processes in drought were enriched with response to abiotic stimulus, response to stress, and defense response (Supplementary Table 4A). Similarly, in salt stress conditions, both tissues (leaf, and root) showed the significant presence of lipoxygenase activity, oxidoreductase activity, and cytokinin dehydrogenase activity, and phosphoric ester hydrolase activity in MF. BP in salt was enriched with the carboxylic acid biosynthetic process, response to stress, response to abiotic stimulus, response to oxidative stress, and cell redox homeostasis (Supplementary Table 4B). In cellular component, apoplast, cell wall, and extracellular region were enriched in drought and cell wall, extracellular matrix, apoplast, cell cortex, and oxygen-evolving complex were enriched in salt stress data.

**FIGURE 4:**
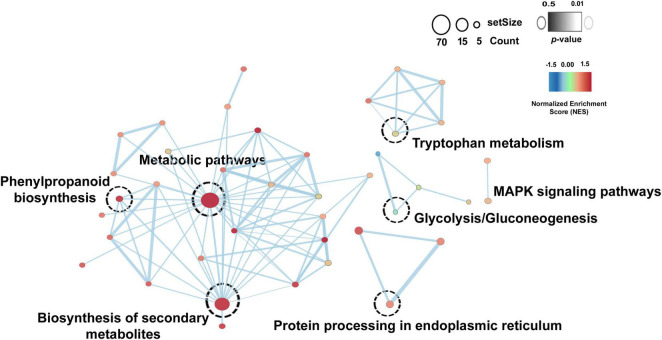
Common enriched pathways in drought and salt dataset. Node color shows the normalized enrichment score (NES) from the core enrichment genes. A positive score (red) shows gene set enrichment at the top of the ranked list, and a negative score (blue) indicates gene set enrichment at the bottom of the ranked list. The color of the border represents the *p*-value of the enriched pathway.

Overall, the Kyoto Encyclopedia of Genes and Genomes (KEGG) pathways enrichment analysis reveals the role of drought stress response in Ubiquinone and other terpenoid-quinone biosynthesis and MAPK signaling pathways (Supplementary Table 5A) and salt stress responses in phenylpropanoid biosynthesis and Isoquinoline alkaloid biosynthesis (Supplementary Table 5B). Based on significant log2fold change (≤ -2 or ≥ 2) and *p*-value ≤ 0.05, *TLP3, TLP5, TLP6, TLP, NCED1, EXLB1, PBS1, PCL1, BT2, TAR4, ITN1, ORG2, WRK54*, and *CAX3* genes were highly upregulated in drought stress condition and *TLP1, TLP, GSTXA, NCS2, AKR1, LAC14, PDR1, ERD10, INV1, HSP70, DOX1, AOC, GPAT3, ATHB7, PER1, ELF4, EXLB1, RAV1, PER30*, and *CIPK6* were highly upregulated in salt stress condition. Overall, this study highlights the key role of these pathways in drought and salt stress responses. Additionally, the common differentially expressed genes (DEGs) were identified in both drought and salt stress conditions, and details of significant DEGs are listed in Supplementary Table 6. By utilizing these genes the common enriched pathways were identified and represented through Cytoscape using the EnrichmentMap module (Figure 4). Biosynthesis of secondary metabolites, metabolic pathways, tryptophan metabolism, MAPK signaling pathways, protein processing in the endoplasmic reticulum, and phenylpropanoid biosynthesis pathways were commonly found in both drought and salt datasets.

### Hub Genes Identification, Functional Enrichment, and Kyoto Encyclopedia of Genes and Genomes Pathways Analysis of Putative Hub Genes

In the co-expression network, the genes that have a high correlation with other genes are known as hub genes. Therefore, a topological feature of a network can be used to know the specific drought and salt stress-responsive genes. Using Cytoscape’s Network analyzer tool, candidate hub nodes were determined using two topological features, betweenness (the fraction of all shortest paths that include a node within a network) and degree (number of connections) calculation. Altogether, we have identified 86 and 61 nodes with a high degree of connectivity and betweenness value and were subsequently referred to as hub genes in the network in drought (Table 1A) and salt stress conditions (Table 1B).

**FIGURE 5:**
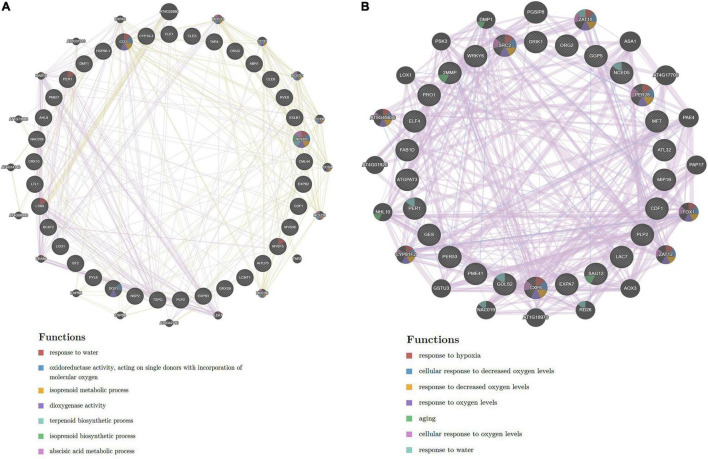
Gene network of hub genes derived from GeneMANIA along with functional enrichment in **(A)** drought and **(B)** salt stress conditions.

**FIGURE 6:**
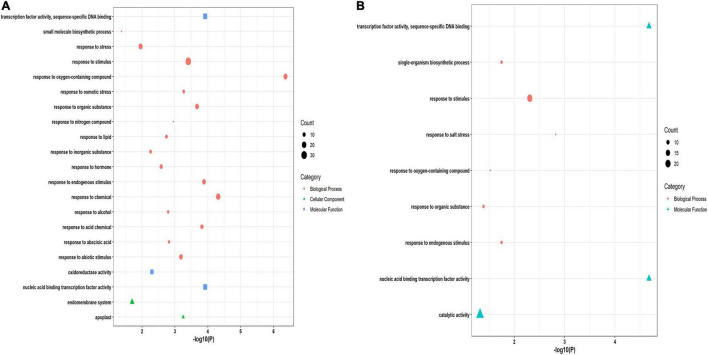
Gene ontology (GO) classification of hub genes in **(A)** drought **(B)** salt stress condition. The x-axis represents significant *P*-values. The y-axis denotes the enriched functions. Bubble size denotes the number of DEGs enriched in the putative functions.

A functional enrichment study of 86 and 61 putative drought and salt stress-responsive hub genes was performed by using GeneMania (GM) webserver and GO analysis. Through the GM webserver, we also predicted interactions between hub genes in the network using *Arabidopsis thaliana* as an additional parameter. The GM outcomes showed their role in response to water, oxidoreductase activity, acting on single donors with incorporation of molecular oxygen, isoprenoid metabolic process, dioxygenase activity, terpenoid biosynthetic process, isoprenoid biosynthetic process, and abscisic acid metabolic process in drought stress conditions (Figure 5A) while response to hypoxia, cellular response to decreased oxygen levels, cellular response to oxygen levels, response to water and aging in salt stress condition (Figure 5B). Furthermore, to confirm the function of GM, gene ontology classification has also been done. The GO analysis showed their role in response to stress, response to stimulus, response to abiotic stimulus, response to abscisic acid, and oxidoreductase activity in drought (Figure 6A). The highest number of genes were involved in response to stimulus, then in response to salt stress, catalytic activity and response to endogenous stimulus in salt stress condition (Figure 6B).

**FIGURE 7:**
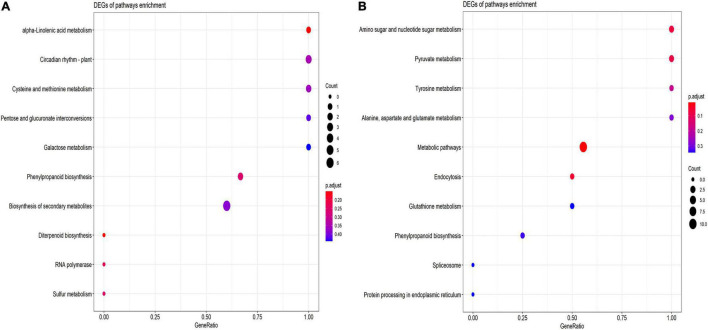
Advanced bubble chart shows putative KEGG pathways in **(A)** drought **(B)** salt stress conditions. The x-axis represents GeneRatio, which is the ratio of the number of DEGs and all annotated genes. The y-axis denotes the enriched pathways. Enrichment significance is shown with color and bubble size denotes the number of DEGs enriched in the pathway.

The KEGG pathways analysis demonstrated the role of drought stress response in phenylpropanoid biosynthesis, biosynthesis of secondary metabolites and diterpenoid biosynthesis, and sulfur metabolism pathways in which the highest number of genes are involved in the biosynthesis of secondary metabolites (Figure 7A) and salt stress, the higher the number of genes is involved in metabolic pathways than in phenylpropanoid biosynthesis and pyruvate metabolism (Figure 7B).

### Expression Profile of Putative Hub Genes

**FIGURE 8:**
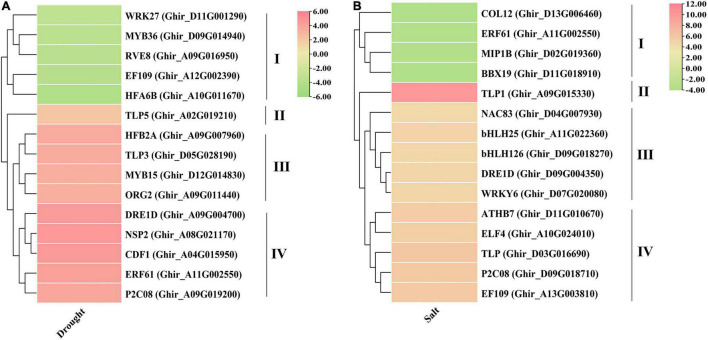
Expression profiles of putative hub genes under **(A)** drought and **(B)** salt stress conditions. These expressions have been shown with the pheatmap package in R using Log2FC values.

Based on degree centrality cutoff ≥ 30 and significant differential values (log2FC ≥ 2 or ≤ −2 with *p*-value ≤ 0.05) the expression profiles were illustrated for identified hub genes in both drought and salt stress conditions. The expression pattern revealed that *NSP2, CDF1, DRE1D, ERF61, P2C08, HFB2A, TLP3, MYB15, ORG2*, and *TLP5* genes have significantly higher expression in drought stress conditions (Figure 8A). All the drought-responsive genes were majorly clustered in four groups, three of them showing upregulated genes, in which the *TLP5* gene does not cluster with another gene. Moreover, *TLP1, TLP, P2C08, EF109, ATHB7, ELF4, bHLH25, DRE1D, WRKY6, bHLH126*, and *NAC83* genes were expressed significantly higher in salt stress (Figure 8B), in which Tubby-like protein 1 (*TLP1*) showed the highest expression (>10-fold) among the identified genes also making the separate cluster from other. These outcomes suggested that TLP might have some specific role in drought and salt stress conditions.

**FIGURE 9:**
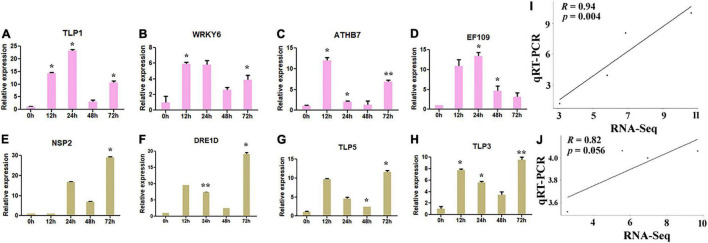
The expression pattern of key hub genes in *G. hirsutum*. qRT-PCR expression pattern of four putative genes in normal (0 h), 12, 24, 48, and 72 h of salt (300 MM) **(A–D)** and drought (20% PEG solutions PEG8000) stress **(E–H)** in cotton. Ubiquitin was used as the loading control. Three biological replicates were used for each experiment. The statistical analysis was performed, using two-tailed Student’s *t*-test. The data are plotted as means ± *SD*. The error bars represent standard deviations. **(I,J)** Correlation analysis against the expression values of qRT-PCR and RNA-Seq data in drought and salt stress conditions. The R represents the Pearson correlation coefficient, respectively. Graph showing a significant positive correlation between qRT-PCR and RNA-Seq data values.

Further, to validate the putative hub genes of *G. hirsutum* in response to drought and salt stresses, the qRT-PCR validation of four key genes in drought (*NSP2, DRE1D, TLP5, and TLP3*) and four in salt (*TLP1, WRKY6, ATHB7, and EF109*) stress conditions was carried out. The corresponding primers are listed in Table 2. The expression of four putative genes was significantly upregulated in salt-stressed conditions, where the majority of the genes showed higher responses at 12, 24, and 72 h (Figures 9A–D) in terms of fold change (FC). *TLP1* showed the highest expression at 24 h (>23 FC) and 12 h (>14 FC) compared with control (Figure 9A). Moreover, *WRKY6* (>5 FC) and EF109 (>10 FC) also showed the highest expression at 12 h and 24 h compared with control (Figures 9B,D) whereas *ATHB7* showed the highest expression at 12 h (>12 FC) in comparison to control (Figure 9C). Likewise, the expression of four putative genes was also upregulated in drought-stressed conditions, where most of the genes showed higher expression at 12 and 72 h (Figures 9E,F), where *NSP2* showed the highest expression at 24 h (>16 FC) and 72 h (>29 FC) (Figure 9E) and *DRE1D, TLP5*, and *TLP3* showed relatively higher expression at 12 and 72 h compared with control (Figures 9F–H). The correlation analysis of key drought (*NSP2, DRE1D, TLP5, and TLP3*) and salt (*TLP1, WRKY6, ATHB7, and EF109*) stress-responsive hub genes also represented a higher positive correlation between transcriptome and qRT-PCR dataset (Figures 9I,J). Altogether, the differential responses of these putative hub genes in salt and drought stresses suggest that these genes may confer to resist the abiotic stresses in cotton.

**TABLE 2:** A list of primers used for validation in qRT-PCR.

Salt stress	Forward primer	Reverse primer
TLP1 (Ghir_A09G015330.1)	GTTTTAACTTGCCGCTTTCG	CGGGTGAACCATATTGACCT
WRKY6 (Ghir_D07G020080.1)	ACGGATGAACACCGAGAATC	CGGCACAATTAGTCCTCCAT
ATHB7 (Ghir_D11G010670.1)	GCAGTGTTGCGGACAAGTTA	TGCTCATAAGGTTGGGCTCT
EF109 (Ghir_A13G003810.1)	GCAGAAGCCCATGAAAAGAC	GAAGTCACCAGGGAAACGAA
**Drought stress**	**Forward primer**	**Reverse primer**
NSP2 (Ghir_A08G021170.1)	TCCATCCCTGCCTATGCTAC	CAACTGCCATCCTCTTCCTC
DRE1D (Ghir_A09G004700.1)	CGATTCTGGGTCTGTTTCGT	TTAGGCTCCTCCACGGTAAA
TLP5 (Ghir_A02G019210.1)	CAACTCGGGATTTCATCAGG	AAGGGTGCTTGGTCATGTTC
TLP3 (Ghir_D05G028190.1)	GCTCTGATGTTGCTGGTGAA	GAGCAGTAAAACGGGCAGAC
Ubiquitin (Internal control)	GAAGCAGCTCGAGGATGGAA	CCACGGAGACGGAGGACAA

### Identification of *Cis*-Regulatory Elements of Putative Hub Genes

**FIGURE 10:**
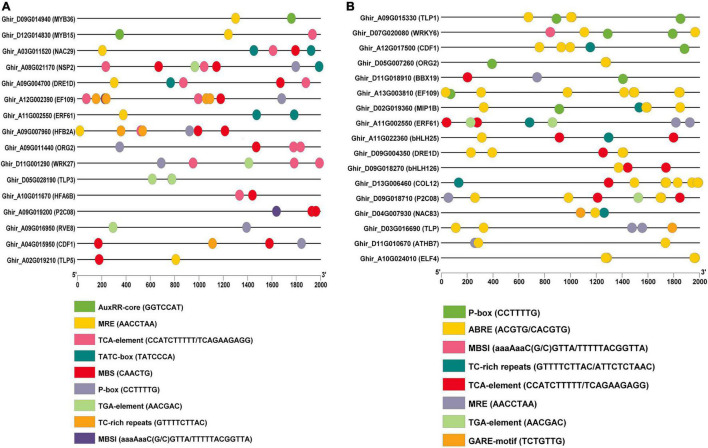
Illustration of *cis*-regulatory elements of putative hub genes in **(A)** drought and **(B)** salt conditions. The micro-parts in diverse colors are the sequence of the putative elements.

The functions of highly expressed hub genes in the expression profiles were further confirmed through *cis*-regulatory elements analysis. We identified the binding frequency of *cis*-regulatory elements in 2,000 kb upstream sequences. In drought stress, *TLP5, NSP2, DRE1D, NAC29, TLP*3, and *HFA6B* comprises the higher number of abiotic stress-responsive elements (Table 3A), and *EF109, MIP1B, ATHB7*, and *TLP* contains the higher number of abiotic stress-responsive elements in salt stress (Table 3B). The upstream region of hub genes showed the abundance of MBS (MYB binding site involved in drought-inducibility) in drought stress conditions (Figure 10A), whereas ABRE (*cis*-acting element involved in the abscisic acid responsiveness) proximal elements were dominant in salt stress conditions (Figure 10B).

**TABLE 3A:** *Cis*-regulatory elements of identified hub genes in (A) drought and (B) salt stress condition.*Cis*-regulatory elements of identified hub genes in drought stress condition.

Gene ids	Gene name	Abiotic stress reponsive elements	Hormone reponsive elements	Others
Ghir_D09G014940	MYB36	4	6	8
Ghir_A03G011520	NAC29	9	7	2
Ghir_A02G019210	TLP5	10	4	5
Ghir_A08G021170	NSP2	9	6	2
Ghir_A09G004700	DRE1D	9	7	8
Ghir_A09G011440	ORG2	7	5	3
Ghir_A12G002390	EF109	7	6	3
Ghir_A09G019200	P2C08	6	8	1
Ghir_D11G001290	WRK27	4	6	0
Ghir_A09G016950	RVE8	3	6	3
Ghir_A11G002550	ERF61	3	6	5
Ghir_A10G011670	HFA6B	8	4	2
Ghir_A04G015950	CDF1	7	4	1
Ghir_D12G014830	MYB15	5	3	4
Ghir_A09G007960	HFB2A	9	2	7
Ghir_D05G028190	TLP3	8	3	7
**Total**		**108**	**83**	**61**

**TABLE 3B:** *Cis*-regulatory elements of identified hub genes in salt stress condition.

Gene ids	Gene name	Abiotic stress reponsive elements	Hormone reponsive elements	Others
Ghir_A09G015330	TLP1	7	5	5
Ghir_D03G016690	TLP	12	4	6
Ghir_D07G020080	WRKY6	10	5	3
Ghir_A10G024010	ELF4	11	5	3
Ghir_A12G017500	CDF1	11	4	0
Ghir_D02G019360	MIP1B	14	5	2
Ghir_D09G004350	DRE1D	11	5	2
Ghir_D09G018710	P2C08	11	7	1
Ghir_D11G010670	ATHB7	13	2	2
Ghir_D13G006460	COL12	11	8	3
Ghir_A11G022360	bHLH25	9	2	1
Ghir_A13G003810	EF109	22	14	5
Ghir_D04G007930	NAC83	10	2	2
Ghir_D05G007260	ORG2	10	4	4
Ghir_D09G018270	bHLH126	5	4	10
Ghir_D11G018910	BBX19	8	2	1
Ghir_A11G002550	ERF61	6	4	1
**Total**		**181**	**82**	**51**

## Discussion

**FIGURE 11:**
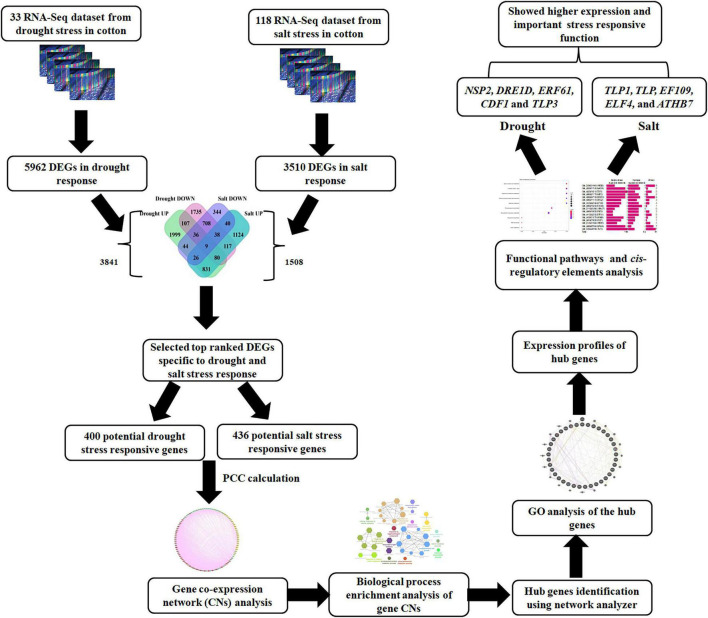
The overall flow chart and strategy for designing and analyzing drought and salt stress-responsive genes.

Cotton is a very important fiber-producing crop that is being cultivated across the globe. It is grown in semi-arid and arid regions where abiotic stresses namely salt and drought are highly prevalent due to which yields get affected. Among these, scarcity of water is a major limitation for the production of the crop. Water deficit conditions can severely lessen the crop yield and adversely influence the biochemical and physiological processes of plants which further lead to the lint yield reduction. The severity of the drought depends on various factors such as moisture storing ability of soils, rainfall amount and distribution, and evaporative demands. In cotton, the growth of the cell is affected by the reduction in turgor pressure due to drought stress. Drought also affects carbohydrate metabolism and photosynthesis directly or indirectly, changes in photosynthesis lead to the reduction of the boll maintenance in cotton (Leakey et al., 2009). The important physiological defense mechanisms to cope with drought stress conditions are root development, stomata closure, photosynthesis, cellular adaptations, JA and ABA hormone production and ROS scavenging have been determined in cotton (Ullah et al., 2017). Apart from water scarcity, soil salinity also gives rise to significant loss (5–9%) of cotton production (Nirmala et al., 2012). Several defense mechanisms such as hormone regulation, membrane stability maintenance, antioxidants generation, and stress proteins induction, and carbon fixation rate have been found to be involved in plant survival under moisture stress conditions. Drought and salt stress conditions enhance the expression level of stress-related transcription factors and genes which further induces several droughts and salt stress-related pathways to stimulate the tolerance in the plant. There are several transcription factors such as *WRKY, NAC, MYB, bHLH, ERF, ORG2, DREB*, and *TLP* which have an important role in DSSR. To know the highly putative genes in drought and stress responses in cotton, comprehensive cotton-DSSR hub genes, and their functional enrichment studies were performed. The overall flow chart and strategy for designing and analyzing drought and salt stress-responsive gene co-expression networks (Figure 11).

By using the transcriptome (RNA-Seq) dataset of different tissues of cotton (*G. hirsutum*) in drought and salt stress conditions, the cotton-DSSR network study was done. Firstly, the GCN with the PCC method was constructed. Further, these GCN were taken for estimating the closeness connectivity, betweenness centrality (Cb), eccentricity, and degree centrality (k) using a network analyzer plugin with a circular layout. Furthermore, the genes are ranked based on betweenness centrality scores and degree connectivity (Figures 3, 4). In drought, SBT, EXPB2, CHS, TLP3, and CML44 genes and in salt stress conditions, WRKY6, ELF4, TLP, EXLB1, AATP1, and PER53 genes were found with a higher degree of connectivity and betweenness centrality scores. Previously, it has been reported that phytosulfokine (PSK) precursor processing by subtilase SBT signaling improves drought stress tolerance in *Arabidopsis* (Stuhrwohldt et al., 2021), expression of expansin-like B2 (EXPB2) enhanced for cell wall extensibility during drought stress conditions in *Solanum pennellii* (Egea et al., 2018). Overexpression of Chalcone synthase (CHS) increases the production of flavonoids, which served as antioxidants in response to different stresses, namely drought, salt, and heavy metal (Chen et al., 2015). In *Arabidopsis*, *TLP3* was found to function in response to ABA (Bao et al., 2014) and overexpression of calmodulin-like (*CML44*) stress-responsive gene from *Solanum habrochaites* improves tolerance to various abiotic stresses (Munir et al., 2016). These genes (SBT, EXPB2, CHS, TLP3, and CML44) were also detected in drought stress conditions. Therefore, putative drought-responsive genes might be crucial for drought stress responses. In salt stress conditions, the *WRKY6* expression was found to be enhanced in response to ROS treatment and it also triggers other plant defense responses and senescence processes (Robatzek and Somssich, 2001; Li et al., 2020). The member of EARLY FLOWERING3 (*ELF*) have an important role in providing tolerant to high NaCl (Sakuraba et al., 2017; Cheng et al., 2020) and Tubby-like protein (TLP) members have been identified to play a crucial role in several abiotic responses such as drought, salt, oxidative (Wardhan et al., 2012; Xu et al., 2016; Li Z. S. et al., 2021). Furthermore, the expression of expansin-like B1 (*EXPB1*) was found to be upregulated by salt, oxidative, and osmotic conditions *Brassica rapa* (Muthusamy et al., 2020). The involvement of these genes (WRKY6, ELF, TLP, and EXLB1) demonstrated an imperative role in salt stress, and these genes, which were also detected in salt stress data, could therefore play an important role in salt stress. For an in-depth understanding of the DSSR network, the biological processes analysis of putative co-expressed responsive genes (Figures 2A,C,E) was carried out with the CluoGo module with a significant *p*-value. These results demonstrated response to water deprivation, cellular response to abiotic stimulus, flavonoid biosynthetic process, phenylpropanoid metabolic process, response to jasmonic acid and trehalose biosynthesis (Figure 2B) biological process in upregulated genes of leaf tissue while, flavonoid biosynthetic process, response to decreased oxygen levels, response to hydrogen peroxide and response to water deprivation (Figure 2D) biological process in upregulated genes of root tissue in drought stress condition. It has been reported that by regulating the homeostasis of ROS, flavonoids improve drought tolerance of maize seedlings (Li B. Z. et al., 2021) and regulation of phenylpropanoid biosynthesis contributes to drought resistance in apple roots by MYB88 and MYB124, responsible for lignin accumulation under stress conditions (Geng et al., 2020). Moreover, the accumulation of jasmonic acid is required for abscisic acid biosynthesis under drought stress conditions in citrus roots (de Ollas et al., 2013) and enhanced trehalose biosynthesis improves yield production under drought stress so there is a positive correlation between high-yield parameters and trehalose overproduction (Joshi et al., 2020). The outcomes of biological processes in salt stress conditions showed cellular response to abiotic stimulus, flavonoid metabolic process, isoprenoid metabolic process, hyperosmotic salinity response, response to abscisic acid, response to decreased oxygen levels, response to extracellular stimulus, and response to jasmonic acid in upregulated genes of root tissue (Figure 3B) whereas, regulation of secondary metabolite biosynthetic process, induced systemic resistance, oxylipin biosynthetic process and cellular response to boron-containing substance levels were upregulated in leaf tissue (Figure 3D). Seed tissue was enriched with malate metabolic process, peptidyl-threonine phosphorylation, basipetalauxin transport, and maintenance of protein location in cells in upregulated genes (Figure 3F). An earlier report stated that *MYB111* is a positive regulator in salt stress response and bioflavonoids addition able to rescue the loss of salt tolerance in *myb111* mutants (Li et al., 2019), and salt stress condition also enhances the biosynthesis of isoprenoid (Zuo et al., 2019), indicating their importance in salt stress condition. Furthermore, oxylipin biosynthesis provides adaptation to plants under salt stress conditions (Savchenko et al., 2014) and TCA cycle intermediates (malate, α-ketoglutarate, aconitate, isocitrate, citrate, and succinate) enhanced during salt stress exposure in the barley cultivar Sahara (salt-sensitive) (Bandehagh and Taylor, 2020). These overall outcomes suggest that high-ranked genes and biological processes of co-expressed networks play a crucial role in drought and salt stress responses at both (root and leaf) tissue levels.

Significantly differentially expressed genes were taken for further biological pathways investigation. The KEGG pathways study revealed the role of interacting genes in Ubiquinone and other terpenoid-quinone biosynthesis and MAPK signaling pathways in drought stress response (Supplementary Table 5A) and phenylpropanoid biosynthesis and Isoquinoline alkaloid biosynthesis in salt stress response (Supplementary Table 5B). Ubiquinone (UQ) is an important prenylquinones that acts as an antioxidant, participates in a plant’s response to stress, and regulates gene expression and cell signal transduction (Liu and Lu, 2016). The expression of genes in terpenoids biosynthesis is affected by drought stress and modulated by transcriptional regulation in trichomes of *Nicotiana tabacum* (Wang et al., 2021). Moreover, enhanced phenylpropanoid concentrations lead to improving the nutritional quality of wheat sprouts under salt stress conditions (Cuong et al., 2020) and increased alkaloid concentration plays an important role in the adaptation to salt conditions (Shi and Gu, 2020). Overall, the KEGG pathway showed that DSSR genes mainly target secondary metabolites-related pathways to provide tolerance against drought and salt stress conditions. Moreover, the common pathways in both drought and salt stress datasets showed that a higher number of genes significantly involved in the biosynthesis of secondary metabolites, metabolic pathways, phenylpropanoid biosynthesis, and tryptophan metabolism pathways (Figure 4). It is reported that tryptophan is a precursor for melatonin and serotonin biosynthesis (Murch et al., 2000), which have a regulatory role in abiotic stress (drought and salt) (Kaur et al., 2015). From outcomes of common enriched pathways, we can hypothesize that the involved genes might have an important role in providing resistance against drought as well as salt stress conditions in cotton.

Based on two topological features, betweenness (the fraction of all shortest paths that include a node within a network) and degree (the number of connections), candidate hub genes were determined using the network analyzer tool. A total of 86 and 61 hub genes were identified with a high degree of connectivity and betweenness value in drought (Table 1A) and salt stress conditions (Table 1B). These identified genes mostly belong to the family of secondary metabolites responsive genes (SMRGs). SMRGs including *MYB15* (Marchev et al., 2020), *NAC29* (Huang et al., 2015; Wang T. T. et al., 2019), *TLP3* (Ibrahim and Jaafar, 2013; Bao et al., 2014), *HFB2A* (Paupiere et al., 2020), *WRKY27*, *OLE16* (Shao et al., 2019), *BBE8* (Daniel et al., 2017), *LEA3* (Zhao et al., 2011), *CHSY* (Dao et al., 2011), *DRE1D* (Agarwal et al., 2017), Malate synthase (de Kraker and Gershenzon, 2011), cycloartenol synthase (Jin et al., 2017) and CASL1 (Taura et al., 2007) in drought enhances secondary metabolite responsive pathways and exert to provide tolerance in the adverse condition in drought stress condition. In salt, several identified genes such as *TLP* (Zare-Hassani et al., 2019; de Jesus-Pires et al., 2020), *WRKY6* (Robatzek and Somssich, 2001; Afrin et al., 2015), MMP-2 (Guo et al., 2019), peroxidase 53 (Novo-Uzal et al., 2014), *bHLH25* (Goossens et al., 2019; Meraj et al., 2020; Yang et al., 2020), malate synthase (Brito et al., 2020), carboxylesterase (Wang et al., 2015; Cao et al., 2019), 1-deoxy-D-xylulose-5-phosphate synthase 2 (Paetzold et al., 2010), NAC (He et al., 2019), *DCS2* (George et al., 2017), *XTH* (Choi et al., 2011; Guerriero et al., 2018), *AtHB-7* (Olsson et al., 2004), *NCED5* (Udomchalothorn et al., 2017), CAS (Shirazi et al., 2019), *ERF* (Meraj et al., 2020), galactinol synthase (Patel et al., 2020), *EXPA7* (Jadamba et al., 2020) and glutathione S-transferase (Nazar et al., 2011; Czerniawski and Bednarek, 2018) are SMRGs exhibited crucial role in providing resistance under salt stress condition. Overall, these putative hub genes might have putative functions in drought and salt stress responses in cotton.

A functional enrichment study of 86 and 61 candidate drought and salt stress-responsive hub genes was performed by using GeneMania (GM) webserver, further their functions were confirmed through gene ontology (GO) classification. The functional enrichment analysis of 86 candidate hub genes in drought stress by GM demonstrated their role in response to water, oxidoreductase activity, acting on single donors with incorporation of molecular oxygen, isoprenoid metabolic process, dioxygenase activity, terpenoid biosynthetic process, and abscisic acid metabolic process (Figure 5A). In rice, oxidoreductase activity implies a positive role in drought tolerance (Huang et al., 2014), in transgenic tobacco, isoprenoid metabolism has an important function during drought (Tattini et al., 2014) and drought stress also affects the gene expression of terpenoids biosynthesis and control its transcriptional regulation in tobacco (Wang et al., 2021). At the molecular level, several physiological processes such as stomatal closure, germination, seed dormancy, and change in gene expression are regulated through ABA, which is a critical hormone, that serves as the core regulator of several stresses (drought, salt, and low temperature) in plants (Ali et al., 2020). These GM functions were confirmed through GO classification, in which molecular functions and biological processes showed similar outcomes such as response to stress, response to stimulus, response to abiotic stimulus, response to abscisic acid, and oxidoreductase activity in drought (Figure 6A). Moreover, in the functional enrichment study of 61 putative hub genes in salt stress conditions, the GM outcomes showed response to hypoxia, cellular response to decreased oxygen levels, cellular response to oxygen levels, response to water, and aging (Figure 5B). GM results revealed the response to decreased oxygen levels and hypoxia, which were caused by several factors such as excess nutrients, phosphorus, water body stratification (layering), and nitrogen, one of the main reasons is saline gradients and also showing the response to water, indicating their important role during salt stress condition. Salt stress-responsive functions of GM were further affirmed through the GO category, the biological process (BP) of GO demonstrating the significant direct role of these candidate genes in salt stress responses also showing significant catalytic activity in molecular function (MF) (Figure 6B). The catalytic activity has been reported to be involved in abiotic stress tolerances by modulating genes (Chin et al., 2019). Overall, a functional enrichment study in drought and salt stress conditions showed a significant critical role in both the stress conditions.

In drought stress, the KEGG pathways are enriched with phenylpropanoid biosynthesis, biosynthesis of secondary metabolites, diterpenoid biosynthesis, and sulfur metabolism pathways (Figure 7A). The phenylpropanoid biosynthetic pathway is triggered under several abiotic stresses such as drought, heavy metals, salinity, high/low temperatures, and ultraviolet radiations, resulting in the accumulation of several phenolic compounds that have the potential to scavenge harmful reactive oxygen species (Sharma et al., 2019). Terpenoid phytoalexins comprise two zealexins and kauralexins families, accumulate in roots in response to both abiotic and biotic stress such as drought, high salinity, *Fusarium verticillioides* infection, and *diabrotica balteata* herbivory. In maize roots, the accumulation of terpenoids is associated with drought tolerance (Vaughan et al., 2015). The previous report stated that sulfur not only acts like other macronutrients but is also imperative during metabolic adaptation reactions to drought stress, indicating sulfur metabolism is required in response to drought stress (Abuelsoud et al., 2016; Ahmad et al., 2016). In salt stress, the KEGG pathways are enriched with metabolic pathways, amino sugar metabolism, phenylpropanoid biosynthesis, and pyruvate metabolism (Figure 7B). The expression profile of phenylpropanoid biosynthetic pathway-related genes and phenylpropanoid compounds levels were investigated and found that enhanced phenylpropanoid concentrations lead to improve nutritional quality of wheat sprouts in the presence of salt treatment (Cuong et al., 2020) and there is a positive correlation of pyruvate pathways under salinity stress condition, as pyruvate pathways get activated under salt stress condition (Che-Othman et al., 2017). Previously it was reported that the amino acid and carbohydrate metabolic pathways play a crucial role during salt stress in *S. lycopersicum* (Zhang et al., 2017). These results demonstrated important signaling pathways that have crucial functions under drought and salt stress conditions.

The transcriptome differential expression profiles of the identified drought and salt stress-responsive hub genes were constructed using significant log2FC and degree centrality values. In drought stress, *NSP2, CDF1, DRE1D, ERF61, P2C08, HFB2A, TLP3, MYB15, ORG2*, and *TLP5* genes show significantly higher expression (Figure 8A). The DEGs in drought were mainly clustered into four groups, in which Group II, III, and IV comprised upregulated genes, while Group I contained downregulated genes. In the upregulated genes, *TLP5* does not group with any other genes, as group II only includes the TLP5 gene. In salt stress, the genes *TLP1, TLP, P2C08, EF109, ATHB7, ELF4, bHLH25, DRE1D, WRKY6, bHLH126*, and *NAC83* show a significantly higher expression (Figure 8B). The DEGs in salt were also clustered into four groups, in which Group II, III, and IV comprised upregulated genes while Group I contained downregulated genes. In the upregulated genes, *TLP1* does not group with other genes, since Group II only includes the *TLP1* gene and showed the highest expression (>10-fold) among the upregulated genes. The *TLP* gene members have been identified in some dicotyledonous and monocotyledonous plants such as maize (Chen et al., 2016), apple (Xu et al., 2016), and Arabidopsis, poplar, rice (Yang et al., 2008). In Arabidopsis, *AtTLP3*, and *AtTLP9* were found to have a redundant effect in response to osmotic stress treatments and ABA (Lai et al., 2004), while *AtTLP9* also responded to salt and drought stress (Lai et al., 2004; Bao et al., 2014). In *Malus domestica*, several *TLP* genes showed higher expression during abiotic stress treatments (Xu et al., 2016). It was found that *CaTLP1* was expressed in response to dehydration stress in *Cicer arietinum*, while its expression led to increased tolerance to salt, drought, and oxidative stress in tobacco (Wardhan et al., 2012). Based on the higher expression of hub genes in the transcriptome dataset, four genes (*NSP2, DRE1D, TLP5, and TLP3*) in drought and four genes (*TLP1, WRKY6, ATHB7, and EF109*) in salt stress were taken for further qRT-PCR validation (Figure 9). The results showed that tubby-like protein (TLP) showed higher expression in both drought and salt stress responses. Further, correlation analysis also supported the significant positive correlation between qRT-PCR and transcriptome data (Figures 9I,J). As per the previous report, the WRKY6 gene helps in the induction of salicylic acid (Robatzek and Somssich, 2001) in *A. thaliana* which has reported an important role in mitigating the adverse effects of salt stress on plants (Ma et al., 2017). The higher expression of *TLP1* in *Cicer arietinum* led to increased tolerance to salt, drought, and oxidative stress in tobacco (Wardhan et al., 2012). Moreover, AtTLP3 member has been found to respond to abiotic stresses (Li Z. S. et al., 2021). The overall outcomes suggested that *NSP2, DRE1D, TLP5, and TLP3* in drought and *TLP1, WRKY6, ATHB7, and* EF109 in salt may be crucial in cotton improvement.

Altogether, these studies suggested that *TLP* gene family members might play an important role in drought as well as in salt stress tolerance and could be suitable targets to protect the cotton from drought and salt stress conditions.

In order to confirm TLP’s functional role and other highly expressed genes in drought and stress conditions, the *cis*-regulatory element analysis was performed. In drought stress, *TLP5, NSP2, DRE1D, NAC29, TLP*3, and *HFA6B* include a higher number of abiotic stress-responsive elements (Table 3A), the upstream region of these putative genes show the abundance of MBS (MYB binding site involved in drought-inducibility) (Figure 10A). This MBS-*cis-element* is required by the *MYB* TF for the gene expression of drought-inducible genes (Kaur et al., 2017). Moreover, the TGA-element (auxin-responsive element) shows dominance in the *TLP3* gene. The plant can regulate stomatal opening, integrate growth and stomatal regulation in drought conditions by utilizing auxin in Arabidopsis (Salehin et al., 2019). In salt stress, *EF109, MIP1B, ATHB7*, and *TLP* contain a higher number of elements that respond to abiotic stress in salt stress (Table 3B), where ABRE elements (*cis*-acting element involved in the abscisic acid responsiveness) were abundant (Figure 10B). Previously it was stated that ABA is a crucial plant hormone with an important role in regulating salt stress responses (Ha et al., 2014), salinity exposure enhances the accumulation of ABA, resulting in increased tolerance (Zhu, 2002). In addition, *TLP1* and *WRKY* comprise the abundance of P-box (gibberellin-responsive element) in their upstream region. Gibberellic acid is one of the essential plant growth regulators that help improve salt tolerance and also reduce the effects of salt stress on plants (Chauhan et al., 2019) and also induces high salinity tolerance in *Pisum sativum* through upregulation of antiporter genes, antioxidants, and secondary metabolites (Ahmad et al., 2021). Altogether, these studies suggested that *NSP2, DRE1D, TLP5, and TLP3* genes in drought and *TLP1, WRKY6, ATHB7, and EF109* genes in salt stress might play an important role in cotton and could be suitable targets to protect the cotton crop from drought and salt stress condition.

## Conclusion

Overall, our integrative transcriptome meta-analysis and its network topology analysis revealed that *NSP2, DRE1D, ERF61, CDF1*, and *TLP3* genes induced strong drought stress responses, accentuated by the increased expression levels and the response of *TLP1, TLP, EF109, ELF4*, and *ATHB7* genes enhanced during salt stress condition. The functional enrichment study of candidate hub genes showed the role of oxidoreductase and dioxygenase activity, response to an abiotic stimulus along with several secondary metabolite responsive genes in drought stress conditions, whereas the response to stimulus and salt, response to hypoxia, and decreased oxygen levels with catalytic activity responsive genes in salt stress condition. Moreover pathways analysis also demonstrating the higher numbers of putative hub genes involved in secondary metabolite responsive pathways that have found an important role in drought and salt stress states. These drought and salt stress responsive functions were further confirmed through the higher expression values of putative genes in transcriptome data and qRT-PCR validation and proximal element studies. These droughts (*NSP2, DRE1D, TLP5, and TLP3*) and salt (*TLP1, WRKY6, ATHB7, and EF109*) stress-responsive hub genes exert tolerance against unfavorable conditions and protect the cotton crop from drought and stress conditions. These protective actions of hub genes might account to reduce the loss of crop productivity and provide resistivity in adverse states.

## Data Availability Statement

The original contributions presented in the study are included in the article/Supplementary Material, further inquiries can be directed to the corresponding author/s.

## Author Contributions

NB carried out the bioinformatics analysis, designed, and drafted the manuscript. SF performed quantitative expression analysis. CM and SB participated to supervise the study. All authors have read and approved the final manuscript.

## Conflict of Interest

The authors declare that the research was conducted in the absence of any commercial or financial relationships that could be construed as a potential conflict of interest.

## Publisher’s Note

All claims expressed in this article are solely those of the authors and do not necessarily represent those of their affiliated organizations, or those of the publisher, the editors and the reviewers. Any product that may be evaluated in this article, or claim that may be made by its manufacturer, is not guaranteed or endorsed by the publisher.

## Footnotes

1
http://www.cytoscape.org

## Supplementary Material

Supplementary Figure 1 Heatmap cluster of expression in different tissues (leaf, root, and seed) under drought and salt stress conditions in cotton using log2FC.

Supplementary Figure 2 Venn diagram showing the number of shared and unique genes in DEGs of drought and salt stress data in the root, leaf, and seed tissues.
