# Supplementary figures and images for: Transcriptome Meta-Analysis Associated Targeting Hub Genes and Pathways of Drought and Salt Stress Responses in Cotton (Gossypium hirsutum): A Network Biology Approach
Source: Front Plant Sci. 2022 Apr 25;13:818472. doi: 10.3389/fpls.2022.818472 (PMC9083274; doi:10.3389/fpls.2022.818472)

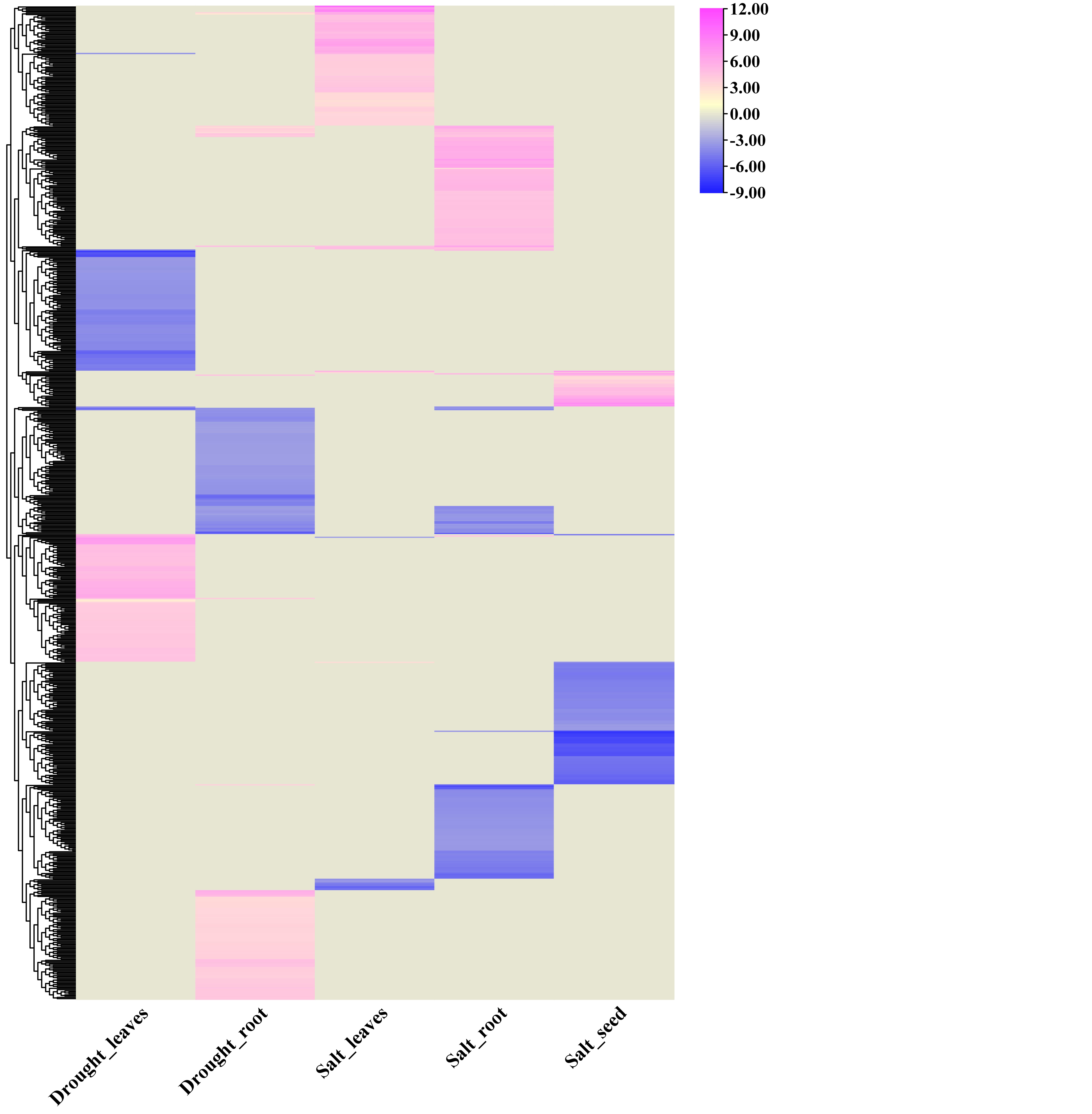

Supplement: Supplementary Figure 1 — Heatmap cluster of expression in different tissues (leaf, root, and seed) under drought and salt stress conditions in cotton using log2FC. [file Image_1.JPEG]

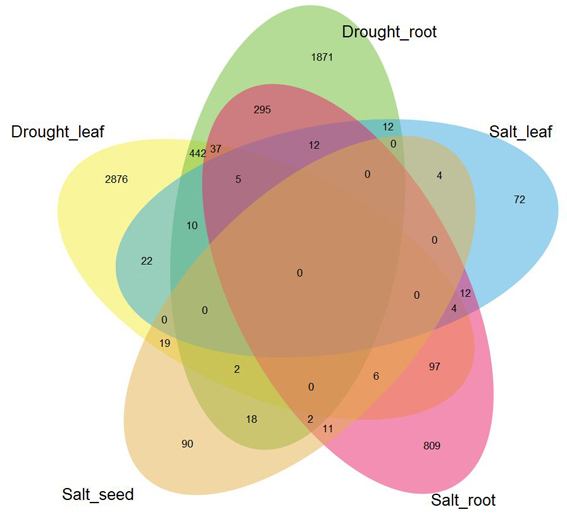

Supplement: Supplementary Figure 2 — Venn diagram showing the number of shared and unique genes in DEGs of drought and salt stress data in the root, leaf, and seed tissues. [file Image_2.JPEG]
